# Supplementary material for: Perceived stress and coping strategies among nursing students towards rejoining college after covid-19 pandemic
Source: PLoS One. 2024 Aug 1;19(8):e0305588. doi: 10.1371/journal.pone.0305588 (PMC11293700; doi:10.1371/journal.pone.0305588)
Supplement: Attachment — Submitted filename: PONE-D-23-24440_reviewer (1).pdf [file pone.0305588.s002.pdf]

PLOS ONE

PERCEIVED STRESS AND COPING STRATEGIES AMONG NURSING STUDENTS TOWARDS REJOINING COLLEGE AFTER COVID-19PANDEMIC

--Manuscript Draft--

**Manuscript Number:**	PONE-D-23-24440
**Article Type:**	Research Article
**Full Title:**	PERCEIVED STRESS AND COPING STRATEGIES AMONG NURSING STUDENTS TOWARDS REJOINING COLLEGE AFTER COVID-19PANDEMIC
**Short Title:**	PERCEIVED STRESS AND COPING STRATEGIES AMONG NURSING STUDENTS TOWARDS REJOINING COLLEGE AFTER COVID-19.
**Corresponding Author:**	Sarmila Koirala, MN, Adult Health Nursing<br>Nepalese Army Institute of Health Sciences College of Nursing<br>Kathmandu, Bagmati NEPAL
**Keywords:**	Key Words: Coping; nursing students; pandemic; Perceived Stress
**Abstract:**	Introduction: COVID-19's devastating and unpredictable global spread has resulted in remarkable global lockdowns and a massive burden on healthcare systems. The global pandemic has proven to be challenging for students in many ways. Following COVID-19, the nursing education that requires students to learn clinical skills and theory has been abruptly discontinued. Nursing students have been worried about poor clinical skill development because a large portion of nursing education involves clinical practice. Because of this, the researcher was motivated to learn how nursing students perceive stress and develop coping mechanisms for returning to nursing colleges following the COVID-19 pandemic.<br><br>Objective: The present study aimed to assess perceived stress and coping strategies among nursing students toward rejoining college after COVID -19 pandemic.<br><br>Methodology: An analytical cross-sectional study was conducted using proportionate stratified simple random sampling technique among nursing students of all nursing colleges affiliated to Purbanchal University in Kathmandu Valley. Altogether, 317 nursing students of bachelor level were selected. Data was collected by using a self-administered questionnaire. Perceived stress was assessed using Perceived Stress Scale (PSS), a five-point Likert scale with ten items, and coping strategies were considered using the Brief COPE Scale, a four-point Likert scale with 28 items.<br>Results: The majority of 71.3 % of the respondents' stress level was moderate, and 28.7% were high. The level of coping among half 51.7% of the respondents was low, and nearly half, 48.3%, had a high coping.There was significant association between level of coping and mother's occupation (p=0.003).The Pearson correlation between perceived stress and coping strategies was moderately positive (r = 0.256, p=0.001).<br><br>Conclusion: The nursing college administration and instructors might contribute to lowering students' stress with effective coping strategies. Encouraging and supporting students through this challenging period by maintaining a safe college environment for rejoining college also can be done.
**Order of Authors:**	Sarmila Koirala, MN, Adult Health Nursing
	Rupa Devi Devi Thapa, MN, Adult Health Nursing
	Sagun Bhandari, MN, Child Health Nursing
	Alisha Rijal, M.Sc. Psychiatric Nursing
	Bhawani Shahi Thakuri, MN, Psychiatric Nursing
	Pooja Gauro, MN, Child Health Nursing
**Additional Information:**	
**Question**	**Response**

Powered by Editorial Manager® and ProduXion Manager® from Aries Systems Corporation

**Financial Disclosure**<br><br>Enter a financial disclosure statement that describes the sources of funding for the work included in this submission. Review the submission guidelines for detailed requirements. View published research articles from *PLOS ONE* for specific examples.<br><br>This statement is required for submission and **will appear in the published article** if the submission is accepted. Please make sure it is accurate.<br><br>**Unfunded studies**<br>Enter: *The author(s) received no specific funding for this work.*<br><br>**Funded studies**<br>Enter a statement with the following details:<br>• Initials of the authors who received each award<br>• Grant numbers awarded to each author<br>• The full name of each funder<br>• URL of each funder website<br>• Did the sponsors or funders play any role in the study design, data collection and analysis, decision to publish, or preparation of the manuscript?<br>• **NO** - Include this sentence at the end of your statement: *The funders had no role in study design, data collection and analysis, decision to publish, or preparation of the manuscript.*<br>• **YES** - Specify the role(s) played.<br><br>* typeset	Authors received grant form University Grant Commission for conducting this research. Fund was not sufficient so some fund was contributed by researchers and completed the study.<br>Authors who received award are:<br>Sarmila Koirala, Sagun Bhandari and Bhawani Shahi Thakuri.<br>Grant awarded is nepali rupees Rs.100,000.<br>Webside of funder is : https://www.ugcnepal.edu.np/<br>The funders had no role in study design, data collection and analysis, decision to publish, or preparation of the manuscript.
**Competing Interests**<br><br>Use the instructions below to enter a competing interest statement for this submission. On behalf of all authors, disclose any competing interests that could be perceived to bias this work—acknowledging all financial support and any other relevant financial or non-financial competing interests.<br><br>This statement is required for submission	Sarmila Koirala : Principal Investigator had role in application for fund, proposal writing, tool selection, data collection, analysis and preparing research report and manuscript.<br>Rupa Devi Thapa: Data collection and preparing research report and manuscript.<br>Sagun Bhandari: Writing proposal and research report.<br>Alisha Rijal: Data collection and analysis<br>Bhawani Shahi Thakuri: Proposal writing, data collection and writing research report.<br>Pooja Gauro: Data collection, analysis and preparation of manuscript.

Powered by Editorial Manager® and ProduXion Manager® from Aries Systems Corporation

and **will appear in the published article** if the submission is accepted. Please make sure it is accurate and that any funding sources listed in your Funding Information later in the submission form are also declared in your Financial Disclosure statement.<br><br>View published research articles from *PLOS ONE* for specific examples.<br><br>**NO authors have competing interests**<br><br>Enter: *The authors have declared that no competing interests exist.*<br><br>**Authors with competing interests**<br><br>Enter competing interest details beginning with this statement:<br><br>*I have read the journal's policy and the authors of this manuscript have the following competing interests: [insert competing interests here]*<br><br>* typeset	
**Ethics Statement**<br><br>Enter an ethics statement for this submission. This statement is required if the study involved:<br><br>• Human participants<br>• Human specimens or tissue<br>• Vertebrate animals or cephalopods<br>• Vertebrate embryos or tissues<br>• Field research<br><br>Write "N/A" if the submission does not require an ethics statement.<br><br>General guidance is provided below. Consult the submission guidelines for detailed instructions. **Make sure that all information entered here is included in the Methods section of the manuscript.**	Firstly, permission letter was taken from all 11 colleges for data collection and ethical approval was taken from Institutional Review Board (IRB) of Nepal Health Research Council(NHRC) for ethical clearance. Approval Number for this study is 657/2021P and Reference no 1302. Written informed consent was taken from each respondent after explaining the purpose of the study. Confidentiality of the information of the respondents was maintained by not disclosing the information and by using information for the purpose of the study. Respondents dignity was maintained by giving right to reject or discontinue from the study at any time without any penalty.

**Format for specific study types**<br><br>**Human Subject Research (involving human participants and/or tissue)**<br>• Give the name of the institutional review board or ethics committee that approved the study<br>• Include the approval number and/or a statement indicating approval of this research<br>• Indicate the form of consent obtained (written/oral) or the reason that consent was not obtained (e.g. the data were analyzed anonymously)<br><br>**Animal Research (involving vertebrate animals, embryos or tissues)**<br>• Provide the name of the Institutional Animal Care and Use Committee (IACUC) or other relevant ethics board that reviewed the study protocol, and indicate whether they approved this research or granted a formal waiver of ethical approval<br>• Include an approval number if one was obtained<br>• If the study involved *non-human primates*, add *additional details* about animal welfare and steps taken to ameliorate suffering<br>• If anesthesia, euthanasia, or any kind of animal sacrifice is part of the study, include briefly which substances and/or methods were applied<br><br>**Field Research**<br><br>Include the following details if this study involves the collection of plant, animal, or other materials from a natural setting:<br>• Field permit number<br>• Name of the institution or relevant body that granted permission	
**Data Availability**<br><br>Authors are required to make all data underlying the findings described fully available, without restriction, and from the time of publication. PLOS allows rare exceptions to address legal and ethical concerns. See the PLOS Data Policy and FAQ for detailed information.	Yes - all data are fully available without restriction

A Data Availability Statement describing where the data can be found is required at submission. Your answers to this question constitute the Data Availability Statement and **will be published in the article**, if accepted.<br><br>**Important:** Stating 'data available on request from the author' is not sufficient. If your data are only available upon request, select 'No' for the first question and explain your exceptional situation in the text box.<br><br>Do the authors confirm that all data underlying the findings described in their manuscript are fully available without restriction?	
**Describe where the data may be found in full sentences. If you are copying our sample text, replace any instances of XXX with the appropriate details.**<br><br>• If the data are **held or will be held in a public repository**, include URLs, accession numbers or DOIs. If this information will only be available after acceptance, indicate this by ticking the box below. For example: *All XXX files are available from the XXX database (accession number(s) XXX, XXX.*).<br>• If the data are all contained **within the manuscript and/or Supporting Information files**, enter the following: *All relevant data are within the manuscript and its Supporting Information files.*<br>• If neither of these applies but you are able to provide **details of access elsewhere**, with or without limitations, please do so. For example:<br><br>*Data cannot be shared publicly because of [XXX]. Data are available from the XXX Institutional Data Access / Ethics Committee (contact via XXX) for researchers who meet the criteria for access to confidential data.*<br><br>*The data underlying the results presented in the study are available from (include the name of the third party*	All relevant data are within the manuscript and its Supporting Information files.

and contact information or URL).<br>• This text is appropriate if the data are owned by a third party and authors do not have permission to share the data.<br><br>* typeset	
Additional data availability information:	Tick here if your circumstances are not covered by the questions above and you need the journal’s help to make your data available.

Manuscript Click here to access/download;Manuscript;Manuscript (Draft, 2023).docx

PERCEIVED STRESS AND COPING STRATEGIES AMONG NURSING STUDENTS TOWARDS REJOINING COLLEGE AFTER COVID-19 PANDEMIC

1.Principal Investigator: Sarmila Koirala

Designation: Assistant Professor, Email Id: sarmilakoirala45@gmail.com

Affiliated Organization: Nepalese Army Institute of Health Sciences,College of Nursing, Sanobharyang, Bhandarkhal, Kathmandu

2. Co- author: Rupa Devi Thapa

Designation: HoD Nursing, Email Id: rupadthapa82@gmail.com

Affiliated Organization: Yeti Health Science Academy, Kantimarga, Maharajgunj, Kathmandu

3. Co- author: Sagun Bhandari

Designation: BSN Program Co-ordinator, Email Id: sagunbhandaribk@gmail.com

Affiliated Organization: Yeti Health Science Academy, Kantimarga, Maharajgunj, Kathmandu 3.

4. Co- author: Alisha Rijal

Designation: Assistant Professor, Email Id: alisha.rijal2016@gmail.com

Affiliated Organization: Yeti Health Science Academy, Kantimarga, Maharajgunj, Kathmandu

5. Co- author: Bhawani Shahi Thakuri

Designation: Lecturer, Email Id: bhawanishahi22@gmail.com

Affiliated Organization: Kathmandu Model Hospital, School of Nursing, Swayambhu, Kathmandu

6. Co- author: Pooja Gauro

Designation: Lecturer, Email Id: pooja.gauro@gmail.com

Affiliated Organization: Asian College for Advance Studies, Satdobato ,Lalitpur

ABSTRACT

Introduction: COVID-19's devastating and unpredictable global spread has resulted in remarkable global lockdowns and a massive burden on healthcare systems. The global pandemic has proven to be challenging for students in many ways. Following COVID-19, the nursing education that requires students to learn clinical skills and theory has been abruptly discontinued. Nursing students have been worried about poor clinical skill development because a large portion of nursing education involves clinical practice. Because of this, the researcher was motivated to learn how nursing students perceive stress and develop coping mechanisms for returning to nursing colleges following the COVID-19 pandemic.

Objective: The present study aimed to assess perceived stress and coping strategies among nursing students toward rejoining college after COVID -19 pandemic.

Methodology: An analytical cross-sectional study was conducted using proportionate stratified simple random sampling technique among nursing students of all nursing colleges affiliated to Purbanchal University in Kathmandu Valley. Altogether, 317 nursing students of bachelor level were selected. Data was collected by using a self-administered questionnaire. Perceived stress was assessed using Perceived Stress Scale (PSS), a five-point Likert scale with ten items, and coping strategies were considered using the Brief COPE Scale, a four-point Likert scale with 28 items.

Results: The majority of 71.3 % of the respondents' stress level was moderate, and 28.7% were high. The level of coping among half 51.7% of the respondents was low, and nearly half, 48.3%, had a high coping.There was significant association between level of coping and mother's occupation (p=0.003).The Pearson correlation between perceived stress and coping strategies was moderately positive (r = 0.256, p=0.001).

Conclusion: The nursing college administration and instructors might contribute to lowering students' stress with effective coping strategies. Encouraging and supporting students through

this challenging period by maintaining a safe college environment for rejoining college also can be done.

Key Words: Coping, Nursing Students, Pandemic, : Perceived Stress

1. INTRODUCTION

COVID-19 was declared as pandemic by the World Health Organization (WHO) on March 11, 2020. The disease is caused by the new coronavirus SARS-CoV-2, which quickly spread across the World. The virus was identified initially in Wuhan, China, in December 2019 and was defined in early January. Public Health Emergency of International Concern was announced on January 30, 2020. COVID-19's devastating and unpredictable global spread has resulted in global lockdowns and a massive burden on healthcare systems[1].

In Nepal the first case of COVID-19 was identified on January 23, 2020, on a Nepali student who had recently returned from Wuhan, China[2]. After no new cases were recorded in February 2020, a 19-year-old girl who had returned from France on March 17 was diagnosed with COVID-19 on March 23, 2020 [3]. Nepal also closed all schools to prevent disease at the earliest possible stage, sealed its international borders, and implemented a nationwide lockdown until June 14, 2020.

After the lockdown, colleges and schools continued education by distance education. Youngsters started worrying about COVID-19, the duration of the pandemic, social distancing, which caused them to experience stress [4] .Many universities implemented preventive measures, including closing colleges, canceling classes, transitioning to online-based teaching/learning, examinations and postponing practical based learning. No one could predict how long such measures would continue and whether they will be able to resume their activities as before. Such disruptions can exert extra additional pressures which adversely affect students' mental health, increasing stress, anxiety, and depression [5,6].

Considering the pandemic's magnitude and rapid spread, the increased worry and stress in the general population, students, and healthcare workers are understandable [7,8]. The disease significantly impacts mental health, causing people to experience various degrees of emotional problems [9]. College students' mental health and unhealthy behavior patterns took important place in professional and public discussions. College students often faced situations that can be risky in terms of psychiatric disorders [10].The alarming prevalence of 80% of perceived stress among

medical college students, including nursing, the use of different coping strategies, and their recommendation for the need to stress management programs [11] contribute to the importance of research in this issue.

It can be noticed that individuals' mental illness is an economic burden, especially for their families, both in terms of treatment costs and reducing productivity [12]. Low or moderate stress levels might enhance students' motivation, leading to greater effort while studying and achieving their goals. Conversely, high-stress levels can negatively influence students, leading to anxiety and depression, affecting students' health as well as academic performance [13].

The interruption of education for nursing students has been unexpected from students. In addition, the clinical practice of nursing students in hospitals has also been discontinued. Because much of nursing education consists of clinical practice, students may have been concerned about inadequate clinical skill development. Also, the application skills are insufficient, so the uncertainty of when, where and how to do the compensatory practicum to eliminate inadequacy could stress nursing students [14.] Along with this students were scared to resume college and their practicum due to spread and emergence of new variants.

In the US 71% of students reported increased stress due to COVID-19 [15]. In Spain, approximately half of the students, 47.92% experienced a moderate level of stress [16.] Likewise, Turkish nursing students experienced a moderate level of stress, with a perceived stress score of 31.69 ± 6.91 [14]. Saudi, nursing students experience a high-stress level [17]. Similarly, Indian nursing students showed a moderate level of stress during COVID-19, with a maximum mean perceived stress score 22.56 [18] . In Nepal, 84.1% of nursing students perceived a moderate stress level [19].

Coping mechanisms are also essential to deal with nursing students' daily stress. Longitudinal studies have shown that stress levels in nursing students may increase or decrease during their educational training, depending on their coping behavior strategies. Besides these differences, coping strategies vary according to the individual's characteristics and the context where the stressors are found [20]. Problem-solving strategies have been identified as one of the best ways to cope with stress. Conversely, emotional-based coping strategies appear to be the least influential [21]. Nepalese nursing students had a moderate coping strategy (63.3%), and adaptive coping strategies were highly used [19]. However, the direction of the relationship between emotional responses and coping strategies is not clear, and the connection is not always

constant [22]. So, the perceived stress and the coping strategies among nursing students during a significant infectious disease such as COVID-19 needs further research to clarify.

As a nursing teacher, the researcher observed stressful concerns among many nursing students and their parents regarding the completion of the academic year. The stress is both on theoretical and practical course completion. Currently, students are attending online classes, which are gradually becoming their new normal. They express not having the will to resume physical classes and clinical posting due to fear of COVID-19 infection and getting habituated to online courses over time. Due to the mandatory rule for fulfilling the practical requirement of nursing education, the students must rejoin physical classes and clinical postings sooner or later.

Therefore the researcher is interested and aimed to find out perceived stress and coping strategies among nursing students towards rejoining college after the COVID-19 pandemic. This will provide evidence to make informed strategies to facilitate enabling environment to motivate students to rejoin physical classes and clinical postings. The study might help nursing college administration and instructors identify stress among students and formulate the protocol for coping strategies which might ultimately improve the quality of nursing education and produce productive students. The findings of the study might be used as a reference point for further research in the future.

2. RESEARCH METHODS

2.1 Study Design

A descriptive, analytical study design was used to assess perceived stress and coping strategies among nursing students towards rejoining college after COVID -19 pandemic.

2.2 Study Setting and Population

This study was conducted in all nursing colleges having Bachelor of Science in Nursing (BSN) and Post Basic Bachelor of Nursing Science (PBNS) Programs affiliated to Purbanchal University located at Kathmandu Valley. There were altogether 11 nursing colleges which have both BSN and PBNS program in Kathmandu Valley. The population of the study was the bachelor level nursing students. The total study population was (N= 1476).Students studying in 2nd and 3rdyear of PBNS and students of 2nd, 3rd and 4th year of BSN program in nursing colleges located at Kathmandu Valley.

2.3 Sampling

Probability, proportionate stratified sampling technique was used for the study. Sample size was calculated by using Cochran's formula .

The Cochran formula is;

Sample size ($n°$) = Z^2pq/d^2. Where Z = 1.96 at 95% level of significance

p (Prevalence of perceived stress) =35.9 % = 0.359 [23] .

q = 1-p = 1-0.359 = 0.641, d = Sample error i.e. 5%= 0.05, N= Target population=1476

By formula, sample size (n^o) = $\frac{(1.96)^2 \times 0.359 \times 0.641}{0.05 \times 0.05} = \frac{3.8416 \times 0.359 \times 0.641}{0.0025} = \frac{0.95694256}{0.0025}$

=353.61

Since population is finite

Sample size (n) = $n°/1 + (n° - 1)/N$

=353.61/1+ (353.61-1)/1476

=287.48

For 10% (non- respondent) = 287.48+28.7 = 317

Total sample size was 317.

Proportionate stratified sampling was used to select the sample from 11 nursing colleges.

n=317

S.N.	Name of College	Total population	Calculation	Required Sample
1.	Asian College for Advanced Studies	BN (38+39=77) BSC (20+20+20=60) =137	317/1476*137	30
2.	B&B Medical Institute	BN (36+40=76) BSC (20+20+20=60) =136	317/1476*136	29
3.	Yeti Health Science Academy	BN (40+39=79) BSC (20+19+19=58) =137	317/1476*137	30
4.	Kantipur Academy of Health Science	BN (38+38=76) BSC (20+20+18=58)	317/1476*134	29

		=134		
5.	Hope International College	BN (33+21=54)<br>BSC (20+20+20=60)<br>=114	317/1476*114	24
6.	Norvic Institute of Nursing Education	BN (38+39=77)<br>BSC (30+29+30=89)<br>=166	317/1476*166	35
7.	Everest College of Nursing	BN (36)<br>BSC (20+20=40)<br>=76	317/1476*76	16
8.	Kathmandu Model College	BN (39+40=79)<br>BSC (20+19+19=58)<br>=137	317/1476*137	30
9.	Om Health Campus	BN (40+39=79)<br>BSC (30+30+30=90)<br>=169	317/1476*169	36
10.	Nepal Institute of Health Science	BN (38+39=77)<br>BSC (20+20+18=58)<br>=135	317/1476*135	29
11.	Nagarik College of Health Science	BN (40+38=78)<br>BSC (18+19+20=57)<br>=135	317/1476*135	29
**Total**		**BN (709)+BSC(767)**<br>**=1476**		**317**

2.4 Research Instrument

Semi structured questionnaire developed. Standard tool, Perceived Stress Scale (PSS) developed by Cohen, Kamarck, & Mermelstein (1994) [24] was used to assess perceived stress among nursing students and Brief-COPE Scale developed by Carver (1997) [25] was used to find out the coping strategies adapted by nursing students. This instrument was divided into three parts: Part I- Questions related to socio-demographic and profession related information. Part II - Perceived Stress Scale (PSS). It is 5 point Likert scale ranging from 0-4, consisting 10 items with maximum possible score of 40. Part III- Brief-COPE Scale. It is 4 point Likert scale ranging

from1-4 with 14 sub scales. Each subscale consists of 2 items where total items are 28 with maximum possible score of 112.

2.5 Ethical Considerations

Firstly, permission letter was taken from all 11 colleges for data collection and ethical approval was taken from Institutional Review Board (IRB) of Nepal Health Research Council for ethical clearance. Approval Number for this study is 657/2021P and Reference no 1302. Written informed consent was taken from each respondent after explaining the purpose of the study. Confidentiality of the information of the respondents was maintained by not disclosing the information and by using information for the purpose of the study. Respondents dignity was maintained by giving right to reject or discontinue from the study at any time without any penalty.

2.6 Data Collection Procedure

Firstly respondents were requested to sign informed consent by explaining the purposes of study. Data was collected through self administered questionnaire form which took about 30 minutes. Data was collected from 21st December 2021 to 11th September 2022. At the end of the data collection, questionnaires was checked for its completeness and accuracy.

2.7 Data Analysis

The data was edited, organized, coded and entered in Statistical Package for Social Science (SPSS) version 20. Data was analyzed by using descriptive and appropriate inferential statistics that is chi-square test, Pearson correlation.

3. RESULTS AND DISCUSSION

The results are presented in two parts. Part I includes descriptive analysis and part II includes inferential analysis.

Part I

TABLE 3.1: Socio-demographic Information of the Respondents

n = 317

Variables	Frequency	Percentage
**Age Group**		
Below 19 years	7	2.2
20 – 24 years	235	74.1
25 – 29	65	20.5
30 – 34	9	2.8
35 years and above	1	0.3
Mean± SD=22.9 ±2.8, Min=19 years Max= 36years		
**Ethnicity**		
Dalit	4	1.3
Janajati	133	42
Madhesi	7	2.2
Brahmin/Chettri	171	53.9
Others	2	0.6
**Religion**		
Hindu	258	81.4
Buddhist	45	14.2
Christian	12	3.8
Others	2	0.6
**Marital Status**		
Married	53	16.7
Unmarried	264	83.2
**Living Arrangements**		
Home	256	80.8
Hostel	26	8.2
Other's	35	11
**Type of Family**		
Joint	70	21.1
Nuclear	232	73.2
Extended	15	4.7

Table 1 reveals the socio-demographic information of the respondents. The respondent's age ranged from 19-36 years with the mean age of 22.9±2.8. Majority of the respondents (74.1%) belonged to age group 20-24 years. Most of the respondents' ethnicity belonged to Brahmin/Chettri (53.9 %) and Janajati (42%) respectively. Majority of the respondents were

hindu (81.4%). Almost 83.2 % of the respondents were unmarried and most of them lived in home (80.8%) and 73.2% belonged to nuclear family.

TABLE 3.2: Family Related Information of the Respondents

n = 317

Variables	Frequency	Percentage
**Family Income**		
Below 20,000	15	4.7
20,001-30,000	35	11
30,001-40,000	45	14.2
40,001-50,000	65	20.5
50,001 and above	157	49.5
**Father's Education**		
Unable to read and write	2	0.6
Can just read and write	18	5.7
Primary level	41	12.9
Secondary level	92	29
Higher Secondary	88	27.8
Bachelor	72	22.7
Others	4	1.3
**Mother's Education**		
Unable to read and write	17	5.4
Can just read and write	56	17.7
Primary level	63	19.9
Secondary level	91	28.7
Higher Secondary	60	18.9
Bachelor	27	8.5
Others	3	0.9
**Occupation of Father**		
Agriculture	39	12.3
Sales &services/ professional	97	30.6
Business	165	52.1
Others	16	5

**Occupation of Mother**		
House wife	213	67.2
Agriculture	23	7.3
Sales &services/ professional	74	23.3
Other's	7	2.2

Table 2 states the family related information of the respondents. Almost half (49.5%) of the respondent's monthly family income was above NRs, 50000. Approximately 27.8 % of the father's education was higher secondary whereas 28.7 % of the mother's education was secondary level. Almost half the respondent's fathers did some kind of business where as 67.2 % of the mother's occupation was house wife.

TABLE 3.3: Professional Characteristics Related Information of Respondents

n = 317

Variables	Frequency	Percentage
**Academic Institution**		
Kantipur Academy of Health Sciences	29	9.1
Asian College for Advanced Studies	30	9.5
B&B Medical Institute	29	9.1
Nepal Institute of Health Sciences	29	9.1
Hope International College	24	7.6
Nagarik College of Health Science	29	9.1
Om Health Campus	36	11.4
Norvic Institute of Nursing Education	35	11
Everest College of Nursing	16	5
Yeti Health Science Academy	30	9.5
Kathmandu Model College	30	9.5
**Program**		
PBBN	137	43.2
BSc. Nursing	180	56.8
**Academic Year**		
Second	88	27.8
Third	150	47.3
Fourth	79	24.9
**Work during Studies**		
Yes	102	32.2
No	215	67.8
**Previous Exposure to Pandemic**		
No	317	100
**Received Support during Pandemic**		
Yes	168	53
No	149	47

Table 3 states the professional characteristics of the respondents. More than half (56.8%) of the respondents were studying Bachelor of Science in Nursing. Near about half of the them (47.3)

studied in third year. Almost 32.2 % of them were involved in work activities. None of them were exposed in any kinds of pandemic before. More than half of the respondent received some kind of support during pandemic.

TABLE 3.4: Descriptive Analysis of Perceived Stress Scale Scores and Brief Coping Scale Scores of the Respondents

n = 317

**Characteristics**	**Minimum to Maximum Scores**	**Obtained Scores**									
		**Score Range**	$\bar{x}$	***SD***	**Mean %**	**Skewness**	**SE of Skewness**	**Z** $_{\text{Skewness}}$	**Kurtosis**	**SE of Kurtosis**	**Z** $_{\text{Kurtosis}}$
**Perceived Stress**	0-40	14-40	22.95	8.09	57.37	1.120	0.137	0.38	-0.184	0.273	-0.67
**Brief Coping**	28-112	28-105	65.55	12.45	58.52	0.145	0.137	1.05	0.718	0.273	2.63
Problem Focused	8-32	8-32	21.69	4.62	67.78	-0.295	0.137	-2.15	-0.275	0.273	-1.07
Emotion Focused	12-48	12-47	27.90	6.29	58.32	0.335	0.137	2.44	0.354	0.273	0.20
Avoidant	8-32	8-29	15.95	4.05	49.84	0.667	0.137	4.86	0.422	0.273	1.54

Table 4 reveals that the perceived stress score ranged from 14 to 40 with a mean of 22.95 (*SD* = 8.09). The perceived stress scale's absolute skew value was lower than 2 and absolute kurtosis value lower than 7. The brief coping score ranged from 28-105 with a Mean±SD of 65.55±*12.45*. The scores for problem focused ranged from 8 to 32 with Mean±SD of 21.69± 4.62, emotion focusedranged from 12 to 47with Mean±SD of 27.9± 6.29and avoidant coping ranged from 8 to 29with Mean±SD of 15.95± 4.05 which states that during stress student mostly adopted problem focused coping strategies with mean percentage of 67.78.

TABLE 3.5: Level of Perceived Stress of the Respondents

Level of Perceived Stress	Frequency	Percentage
Moderate Stress	226	71.3
High Stress	91	28.7
**Total**	**317**	**100**

n = 317

Table 5, reveals the level of perceived stress among the respondents. Majority (71.3%) of the respondents has moderate and almost one third (28.7 %) of the respondents has high stress.

TABLE 3.6: Level of Coping of the Respondents

n=317

Level of Coping	Frequency	Percentage
Low coping	164	51.7
High coping	153	48.3
**Total**	**317**	**100**

Table 6, reveals the level of coping among the respondents. More than half of the respondents (51.7%) have low coping and near about half (48.3%) of the respondents have high coping.

Part II

TABLE 3.7: Association between Level of Stress and Socio-Demographic Information of Respondents

Variables	Level of Stress		χ^2	p-value
	Moderate No. (%)	High No. (%)		
**Age group(in years)**				
<20	26 (72.2)	10(27.8)		
21-25	169 (71.6)	67(28.4)	0.152	0.927*
≥26	31 (68.9)	14(31.1)		
**Ethnicity**				
Janjati	94 (70.7)	39 (29.3)	0.235	0.889*
Brahmin/Chettri	122 (71.3)	49 (28.9)		
Others	10 (76.9)	3 (23.1)		
**Religion**				

Hindu	179 (69.4)	79 (30.6)	2.480	0.115
Other than Hindu	47 (79.7)	12 (20.3)		
**Marital Status**				
Married	35 (66)	18 (34)	0.859	0.354
Unmarried	191 (72.3)	73(27.7)		
**Living Arrangements**				
Home	186 (72.7)	70 (27.3)	1.207	0.272
Other than home	40 (65.6)	21 (34.4)		
**Family Type**				
Nuclear Family	164 (70.7)	68 (29.3)	0.154	0.695
Other than nuclear	62 (72.9)	23 (27.1)		

n=317

Significant level at 0.05 **Likelihood Ratio*

Table 7 shows that there is no significant association between level of stress and socio-demographic information of the respondents.

TABLE 3.8: Association between Level of Stress and Family Related Information

Variables	Level of Stress		χ^2	p-value
	Moderate No. (%)	High No. (%)		
**Income**				
25000 and below	41 (82)	9 (18)		
25001 to 50000	74 (67.3)	36 (32.7)	3.69	0.158
50001 and above	111 (70.7)	46 (29.3)		
**Father's Education**				
Primary level and below	42 (68.9)	19 (31.1)		
Secondary and higher	133 (73.9)	47 (26.1)	1.421	0.491
secondary	51 (67.1)	25 (32.9)		
Bachelor and above				
**Mother's Education**				
Primary level and below	98 (72.1)	38 (27.9)		
Secondary and higher secondary	108 (71.5)	43 (28.5)	0.357	0.837
Bachelor and above	20 (66.7)	10 (33.3)		
**Father's Occupation**				

Agriculture	28 (71.8)	11 (28.2)		
Sales and service/professional	68 (70.1)	29 (29.9)	2.045	0.563*
Business	121 (73.3)	44 (26.7)		
Others	9 (56.3)	7 (43.8)		
**Mother's Occupation**				
Homemaker	157 (73.7)	56 (26.3)	2.142	0.543*
Agriculture	15 (65.2)	8 (34.8)		
Sales and servicel	50 (67.6)	24 (32.4)		
Others	4 (57.1)	3 42.9)		
				**n=317**

Significant Level at 0.05 **Likelihood ratio*

Table 8 shows that there was no significant association between level of stress and family related information of the respondents.

TABLE 3.9: Association between Level of Coping and Socio-demographic Information

Variables	Level of Coping Strategies		χ^2	p-value
	Low<br>No. (%)	High<br>No. (%)		
**Age group(in years)**				
<20	18 (50)	18 (50)		
21-25	121 (51.3)	115 (48.7)	0.327	0.849
≥26	25 (55.6)	20 (44.4)		
**Ethnicity**				
Janjati	73 (54.9)	60 (45.1)	2.868	0.238
Brahmin/Chettri	87 (50.9)	84 (49.1)		
Others	4 (30.8)	9 (69.2)		
**Religion**				
Hindu	130 (50.4)	128 (49.6)	1.008	0.315
Other than Hindu	34 (57.6)	25 (42.4)		
**Marital Status**				
Married	22 (41.5)	31 (58.5)	2.665	0.103
Unmarried	142 (53.8)	122 (46.2)		
**Living Arrangements**				
Home	133 (52)	123 (48)	0.025	0.874
Other than home	31 (50.8)	30 (49.2)		
**Family Type**				
Nuclear Family	43 (50.6)	42 (49.4)	0.061	0.805
Other than nuclear	121 (52.2)	111 (47.8)		

n=317

Significant Level at 0.05

Table 9 shows that there was no significant association between level of coping and socio-demographic variables of the respondents.

TABLE 3.10: Association between Level of Coping and Family related Variables

Variables	Level of Coping Strategies		χ^2	p-value
	Low<br>No. (%)	High<br>No. (%)		
**Income**				
25000 and below	27 (54)	23 (46)		
25001 to 50000	56 (50.9)	54 (49.1)	0.134	0.935
50001 and above	81 (51.6)	76 (48.4)		
**Father's Education**				
Primary level and below	38 (62.3)	23 (37.7)		
Secondary and higher	88 (48.9)	92 (51.1)	3.400	0.183
secondary	38 (50)	38 (50)		
Bachelor and above				
**Mother's Education**				
Primary level and below	69 (50.7)	67 (49.3)		
Secondary and higher	79 (52.3)	72 (47.7)	0.106	0.949
secondary				
Bachelor and above	16 (53.3)	14 (46.7)		
**Father's Occupation**				
Agriculture	16 (41)	23 (59)		
Sales and	48 (49.5)	49 (50.5)		
service/professional			5.097	0.165
Business	94 (57)	71 (43)		
Others	6 (37.5)	10 (62.5)		
**Mother's Occupation**				
Homemaker	123 (57.7)	90 (42.3)		
Agriculture	6 (26.1)	17 (73.9)	14.227	0.003*
Sales and service	30 (40.5)	44 (59.5)		
Others	5 (71.4)	2 (28.6)		

n=317

Significant Level at 0.05 **Likelihood ratio*

Table 10 shows that there was no significant association between level of coping and respondents' family income, parents' education and father's occupation but there was significant association between level of coping and mother's occupation (p=0.003).

TABLE 3.11: Relationship between Perceived Stress and Coping Strategies

n=317

**Variables**	**Correlation**	**p-value**
Stress and Coping Score	0.256	0.001

Significant Level at 0.05

Table 11 depicts the pearson correlation which was calculated to find out bivariate relationship between perceived stress and coping strategies, significant relationship was found between stress and coping among respondents (r = 0.256, p=0.001). The strength of relationship was moderately positive.

Discussion

COVID-19 pandemic sudden onset had disrupted the life of peoples including students in many ways. The pandemic has altered how universities around the world operate. A sudden lockdown in Nepal led to a shift from traditional classroom settings to online learning, impacting university students' academic stress, anxiety, helplessness, and altered quality of life. Student's academic and personal lives have significantly changed due to lockdown, limitations, social isolation, quarantine, and hygiene. The most impacted sector by the COVID-19 pandemic is education. Students were anxious to return to school after the pandemic because of the development of numerous new COVID virus variants.

The range of the perceived stress score in this study was 14–40, with a mean of 22.95±8.09, which is comparable to the Spanish study's mean score of 22.78±8.54 [16], but different from the Turkish study's mean score of 31.69±6.91[14]. Most respondents (71.3%) have moderate stress, while 28.7% have high stress. This is similar to the study conducted in Pune, which found that 82.67% of participants had moderate stress, while 13.35% had high stress [18]. Still, it contrasts with the study conducted in Spain, which found that 47.92% of respondents had moderate stress [16].

The brief coping score ranged from 28-105 with a Mean±SD of 65.55±*12.45*. The scores for problem-focused ranged from 8 to 32 with a Mean±SD of 21.69± 4.62, emotion-focused ranged from 12 to 47 with a Mean±SD of 27.9± 6.29, and avoidant coping ranged from 8 to 29 with a Mean±SD of 15.95± 4, which is in contrast to the findings of Pune where the mean score were 16.57±4.14, 16.87±4.16 and 9.44±2.64 respectively. More than half of the respondents (51.7%) have low coping, near about half (48.3%) of the respondents have high coping, which is in contrast to the study conducted in Pune where only 4.9% of participants had low coping, and 18.5% of participants had high coping [18].The present study's most commonly used coping strategy is problem-focused, similar to those conducted in Spain and Saudi [16,17].

There was no significant association between stress level, socio-demographic information, family-related information, and profession-related information of respondents, which is supported by a study done in Pune. There was no significant association between the level of coping and socio-demographic information and profession-related information of respondents. Still, there was significant association between level of coping and mother's occupation (p=0.003). Still, there was a significant association between the level of dealing with the age of respondents, year of study, and place of residents with a p-value <0.00 [18].

The present study found a significant relationship between stress and coping strategies adopted by respondents ($r = 0.256$, $p=0.001$). The strength of the relationship was moderately positive, which is in contrast to the study conducted in Malaysia, which showed a significant negative correlation ($r= -0.57$, $p<0.05$)[26].

Conclusion

It is concluded that most respondents have moderate stress, and almost one-third of the respondents have high stress. There were no significant influencing variables for the level of stress. More than half of the respondents had low coping, and nearly half had high coping. Among the three coping strategies, mostly adopted coping strategy problem-focused coping. The significant influencing variable for the level of coping was the mother's occupation. A moderately positive relationship was found between stress and coping score, which is statistically significant. This depicts that the respondents with stress have high coping and vice versa. This finding points out a need to introduce stress management programs to help foster healthy coping skills among nursing students.

Conflict of Interest

None

Fund

Funded by University Grants Commission

Acknowledgement

Researcher and the team are grateful to University Grants Commission for providing the fund and Yeti Health Science Academy for allowing to conduct the study. Last but not the least research is thankful to data collection setting and all the participants.

REFERENCES

1. World Health Organization. Clinical management of severe acute respiratory infection when novel coronavirus (2019-nCoV) infection is suspected: interim guidance, 28 January 2020. World Health Organization; 2020.
2. Bastola A, Sah R, Rodriguez-Morales AJ, Lal BK, Jha R, Ojha HC, Shrestha B, Chu DK, Poon LL, Costello A, Morita K. The first 2019 novel coronavirus case in Nepal. The Lancet Infectious Diseases. 2020 Mar 1;20(3):279-80.
3. The Kathmandu Post. 19-year-old woman who returned from France via Qatar test positive for COVID-19. 2020, March 23. https://kathmandupost.com/health/2020/03/23/19-year-old-woman-who-returned-from-france-via-qatar-tests-positive-for-covid-19.
4. Ekiz T, Ilıman E, Dönmez E. Comparison of individuals' health anxiety levels and perception of COVID-19 outbreak control. International Journal of Health Management and Strategies Research 2020 6 (1), 139-154.
5. Neto ML, Almeida HG, Esmeraldo JD, Nobre CB, Pinheiro WR, de Oliveira CR, da Costa Sousa I, Lima OM, Lima NN, Moreira MM, Lima CK. When health professionals look death in the eye: the mental health of professionals who deal daily with the 2019 coronavirus outbreak. Psychiatry research. 2020 Jun 1;288:112972.
6. Naser, A. Y., Dahmash, E. Z., Al-Rousan, R., Alwafi, H., Alrawashdeh, H. M., Ghoul, I., ...&Abuthawabeh, R. (2020). Mental health status of the general population, healthcare professionals, and university students during 2019 coronavirus disease outbreak in Jordan: a cross-sectional study. *medRxiv*.
7. Lai AY, Lee L, Wang MP, Feng Y, Lai TT, Ho LM, Lam VS, Ip MS, Lam TH. Mental health impacts of the COVID-19 pandemic on international university students, related stressors, and coping strategies. Frontiers in Psychiatry. 2020:1082.
8. Usher K, Durkin J, Bhullar N. The COVID-19 pandemic and mental health impacts. International journal of mental health nursing. 2020 Jun;29(3):315.
9. Zhou Y, Wang W, Sun Y, Qian W, Liu Z, Wang R, Qi L, Yang J, Song X, Zhou X, Zeng L. The prevalence and risk factors of psychological disturbances of frontline medical staff in china under the COVID-19 epidemic: workload should be concerned. Journal of affective disorders. 2020 Dec 1;277:510-4.
10. Blanco C, Okuda M, Wright C, Hasin DS, Grant BF, Liu SM, Olfson M. Mental health of college students and their non–college-attending peers: results from the national

epidemiologic study on alcohol and related conditions. Archives of general psychiatry. 2008 Dec 1;65(12):1429-37.

11. Abebe AM, Kebede YG, Mengistu F. Prevalence of stress and associated factors among regular students at Debre Birhan governmental and nongovernmental health science colleges North Showa zone, Amhara region, Ethiopia 2016. Psychiatry journal. 2018 Sep 2;2018.
12. Chavan BS, Das S, Garg R, Puri S, Banavaram AB. Disability and socio-economic impact of mental disorders in the state of Punjab, India: Findings from national mental health survey, 2015–2016. International Journal of Social Psychiatry. 2018 Sep;64(6):589-96.
13. Riley JM, Collins D, Collins J. Nursing students' commitment and the mediating effect of stress. Nurse education today. 2019 May 1;76:172-7.
14. Aslan H, Pekince H. Nursing students' views on the COVID-19 pandemic and their percieved stress levels. Perspectives in psychiatric care. 2021 Apr;57(2):695-701.
15. Son C, Hegde S, Smith A, Wang X, Sasangohar F. Effects of COVID-19 on college students’ mental health in the United States: Interview survey study. Journal of medical internet research. 2020 Sep 3;22(9):e21279.
16. Onieva-Zafra MD, Fernández-Muñoz JJ, Fernández-Martínez E, García-Sánchez FJ, Abreu-Sánchez A, Parra-Fernández ML. Anxiety, perceived stress and coping strategies in nursing students: a cross-sectional, correlational, descriptive study. BMC medical education. 2020 Dec;20:1-9.
17. Alsaqri SH. Stressors and coping strategies of the Saudi nursing students in the clinical training: a cross-sectional study. Education Research International. 2017;2017:1-8.
18. Sheroun D, Wankhar DD, Devrani A, Lissamma PV, Chatterjee K. A study to assess the perceived stress and coping strategies among B. Sc. nursing students of selected colleges in Pune during COVID-19 pandemic lockdown. International Journal of Science and Healthcare Research. 2020 Apr;5(2):280-8.
19. Subba R, Subba HK, Singh JP, Mehta RK. Psychological impact and coping strategies of nursing students during the outbreak of coronavirus disease in Nepal. Asian Journal of Multidisciplinary Studies. 2020 Sep;8(9):23-31.
20. Jimenez C, Navia-Osorio PM, Diaz CV. Stress and health in novice and experienced nursing students. Journal of advanced nursing. 2010 Feb;66(2):442-55.

21. Labrague LJ, McEnroe-Petitte DM, Gloe D, Thomas L, Papathanasiou IV, Tsaras K. A literature review on stress and coping strategies in nursing students. Journal of mental health. 2017 Sep 3;26(5):471-80.
22. Yeung DY, Fung HH. Age differences in coping and emotional responses toward SARS: A longitudinal study of Hong Kong Chinese. Aging and Mental Health. 2007 Sep 1;11(5):579-87.
23. Awoke M, Mamo G, Abdu S, Terefe B. Perceived stress and coping strategies among undergraduate health science students of jimma university amid the COVID-19 outbreak: online cross-sectional survey. Frontiers in psychology. 2021 Mar 30;12:639955.
24. Cohen S, Kamarck T, Mermelstein R. Perceived stress scale. Measuring stress: A guide for health and social scientists. 1994 Jul 15;10(2):1-2.
25. Carver CS. You want to measure coping but your protocol'too long: Consider the brief cope. International journal of behavioral medicine. 1997 Mar;4(1):92-100.
26. Ganesan Y, Talwar P, Fauzan N, Oon YB. A study on stress level and coping strategies among undergraduate students. Journal of Cognitive Sciences and Human Development. 2018 Jun 14;3(2):37-47.
