# Supplementary material for: Perceived stress and coping strategies among nursing students towards rejoining college after covid-19 pandemic
Source: PLoS One. 2024 Aug 1;19(8):e0305588. doi: 10.1371/journal.pone.0305588 (PMC11293700; doi:10.1371/journal.pone.0305588)
Supplement: Attachment — Submitted filename: Response to Author and reviewer.docx [file pone.0305588.s003.docx]

Date: 12/17/2023

To

The Editor

PLOS ONE Journal

**Subject: Rebuttal Letter**

Dear Editor and Reviewers,

First of all I would like to thank you so much for acknowledging our work and guiding us to make it a better manuscript. We all have tried to make corrections as per your suggestions.

1.	The editor of this journal has suggested and made some corrections to make the abstract short but informative. Corrections were made as per instructions. As English is not our native language so the language of manuscript and words picked were not satisfactory we are so happy to see the corrections made.

2.	PLOS ONE style requirements for manuscript submission was reviewed and applied.

3.	Grant information in the funding information section was checked but couldnot include the name of Sagun Bhandari and Bhawani Shahi Thakuri as three of us are fund recipient. In financial disclosure section the amount of fund received was mentioned but the received amount was not sufficient to complete the research so remaining fund was contributed by researchers self. Received amount from UGC: Rs.100,000. Total Expenses Rs.1,62,175.

4.	The authors have declared that no competing interests exist in cover letter.

5.	Funding information from acknowledgement was removed. As there is policy that we should acknowledge the funding agency during publication I have mentioned the name of UGC in acknowledgement section.

6.	Data file was attached.

7.	Reference list was reviewed and corrected.

**Response to Author 1 Recommendations**

1.	Research questions were added in last part of the introduction portion.

2.	Discussion section was revised to relate with existing literature.

**Response to Author 2 Recommendations**

1.	Age section was open ended question in questionnaire. Later range was created after calculating mean value of age and minimum and maximum age. Though the range first was mentioned as ≤ 19 years minimum age was 19 years and there were no one below 19 years so in description less than 19 years was not mentioned. To support this minimum and maximum age was kept in italic words in table with mean age.

2.DOI of some articles were not available and some references were newspaper, report from government so to support this URL/website where these information can be found are attached. Revisions on references were made as per feedback.

I hope we have answered the queries and made corrections as per need. Please let us know if more corrections are needed or still there remains unanswered queries.

Thank You

Assistant Prof

Sarmila Koirala

Nepalese Army Institute of Health Sciences

College of Nursing

Kathmandu ,Nepal
