# Supplementary material for: Perceived stress and coping strategies among nursing students towards rejoining college after covid-19 pandemic
Source: PLoS One. 2024 Aug 1;19(8):e0305588. doi: 10.1371/journal.pone.0305588 (PMC11293700; doi:10.1371/journal.pone.0305588)
Supplement: Attachment — Submitted filename: Response to Author and reviewer.docx [file pone.0305588.s004.docx]

Date: 03/17/2023

To

The Editor

PLOS ONE Journal

**Subject: Rebuttal Letter**

Dear Editor and Reviewers,

First of all I would like to thank you so much for acknowledging our work and guiding us to make it a better manuscript. We all have tried to make corrections as per your suggestions.

1.	Grammatical error was corrected where applicable.

**Response to Author 1 Recommendations**

Thank you so much for appreciation and guidance.

**Response to Author 2 Recommendations**

Thank you so much for appreciation and guidance.

**Response to Author 3 Recommendations**

Thank you so much for constructive feedback.

1. Reviewer suggested to concise introduction in 3 paragraphs .we tried to concise in three but I am so sorry to say that we are able to concise the introduction in 4 paragraphs.
2. In methodology personnel involved in data collection was added in data collection procedure. How calculated sample was achieved and selected was added in sampling section.
3. Duration for assessing perceived stress and coping was added in instrumentation section.
4. There was no operational definition section in this journal guidelines so it was not mentioned earlier. But as per reviewer comment operational definitions were added in 1.4. Operational definitions are defined as per our country's context.
5. Reviewer has suggested to merge some tables and remove table 3.11. We appreciate your comments but want to clear that tables are made according to objectives so to clarify this objectives were added in manuscript. For independent variables there were socio-demographic variables, family related variables and profession related variables so these are shown separately. To identify level of coping and level of stress were separate objectives so these are shown separately and same with association. If it is mandatory to merge tables please let us know we will merge them.
6. In discussion section term significant relationship was replaced with only relationship and this relation was explained in next sentence which was moderately positive.
7. In conclusion section it was made clear how mother's occupation influenced level of coping.

I hope we have answered the queries and made corrections as per need. Please let us know if more corrections are needed or still there remains unanswered queries.

Thank You

Assistant Prof

Sarmila Koirala

Nepalese Army Institute of Health Sciences

College of Nursing

Kathmandu ,Nepal
